# Verification of the Utility of the Standardized *Melissa officinalis* Extract to Control Gut Contractility in Sheep—Ex Vivo Study

**DOI:** 10.3390/ani15050626

**Published:** 2025-02-21

**Authors:** Martyna A. Posłuszny, Magdalena Chłopecka-Słomińska, Sorphon Suor Cherer, Sekhou Cisse, Mohammed el Amine Benarbia, Marta Mendel

**Affiliations:** 1 Division of Pharmacology and Toxicology, Department of Preclinical Sciences, Institute of Veterinary Medicine, Warsaw University of Life Sciences—SGGW, 166, Nowoursynowska St., 02-786 Warsaw, Poland; martyna_posluszny@sggw.edu.pl (M.A.P.); magdalena_chlopecka@sggw.edu.pl (M.C.-S.); 2 Nor-Feed SAS, 3 Rue Amédéo Avogadro, 49070 Beaucouzé, France; sorphon.suor-cherer@norfeed.net (S.S.C.); sekhou.cisse@norfeed.net (S.C.); amine.benarbia@norfeed.net (M.e.A.B.); 3 Joint Lab ANR FeedInTech (FIT: SONAS/Nor-Feed), 49070 Beaucouzé, France

**Keywords:** sheep, *Melissa officinalis*, lemon balm, rosmarinic acid, chlorogenic acid, lithospermic acid, jejunum, colon, contractility

## Abstract

Background: Modern societies and policymakers increasingly emphasize the advancement of organic farming practices and the preservation of rural culture. Sheep farming faces several challenges, including environmental impacts, antibiotic usage, and public concerns regarding animal welfare. The purpose of this study was to investigate the effects of *Melissa officinalis* extract and its principal phenolic acids on the motility of the sheep jejunum and colon, with the goal of assessing their potential as health-promoting feed additives, particularly in animals with dysmotilities. Methods: The trials were performed on isolated jejunum and colon preparations collected from sheep undergoing routine slaughter. The effect of extracts from *Melissa Officinalis* and three major phenolic acids (rosmarinic, chlorogenic, and lithospermic) was evaluated to identify their ability to modify the spontaneous and pharmacologically-induced contractility of circular and longitudinal smooth muscle. Results: Regarding spontaneous contractions: *Melissa officinalis* extract, rosmarinic acid, and lithospermic acid caused a significant decrease in spontaneous contractility in the jejunum and colon, while chlorogenic acid exhibited myocontractile effects except for colon circular preparations where the effect was myorelaxant as in the other acids. In the case of ACh-induced contractions, all acids and the *Melissa officinalis* extract caused mostly a significant decrease in the magnitude of acetylcholine-induced contractions in both longitudinal and circular smooth muscle strips of the colon and jejunum. Conclusions: The findings suggest a predominantly myorelaxant effect of the *Melissa officinalis* extract and its major phenolic acids on the smooth muscle of the sheep jejunum and colon. These results underscore the potential application of the *Melissa officinalis* extract as a feed additive to modulate intestinal motility and address challenges in livestock production.

## Simple Summary

Sheep farming plays a vital role in wool production and rural culture but faces challenges, such as environmental impacts, antibiotic use, and animal welfare concerns. This study investigates the potential of *Melissa officinalis* extract and its main phenolic acids (rosmarinic, chlorogenic, and lithospermic) as health-promoting feed additives by evaluating their effects on sheep intestinal motility. Using isolated jejunum and colon preparations from sheep, this study assessed the impact of the extract and phenolic acids on spontaneous and acetylcholine-induced contractions of smooth muscle under isometric conditions. Results showed that *Melissa officinalis* extract, rosmarinic, and lithospermic acids significantly reduced spontaneous contractility, while chlorogenic acid had mixed effects, including myocontractile activity in some cases. All tested compounds decreased acetylcholine-induced contractions. The findings suggest that *Melissa officinalis* extract and its phenolic acids have a myorelaxant effect, highlighting their potential to modulate intestinal motility and serve as a symptomatic treatment for diarrhea-related conditions in sheep.

## 1. Introduction

Sheep farming is a vital production sector not only for wool but for meat [1]. Currently, organic farming has become more important; it ensures healthy farming and healthy food for today and tomorrow by protecting soil, water, and climate, promoting biodiversity, and not contaminating the environment with chemical inputs or genetic engineering [2]. Antimicrobials are essential in animal health programs, but their use has been scrutinized because of the rise of antimicrobial resistance globally [3]. Ensuring the safety, health, and overall well-being of animals raised for food is both an ethical obligation and a critical component of providing safe food products; as such, the number of animals raised without antibiotics is growing worldwide [4]. To meet the market’s needs, sheep farming has to be efficient, relatively cheap, and cost-effective, but also provide safe, ecologically friendly, and good quality meat and wool. With the European Union ban on antibiotic growth promoters, previously eliminated problems have started to reappear [5]. However, the rise of infection rates may lead to the increased therapeutical use of antibiotics, which is opposite to the efforts to reduce antimicrobials in animals. It demonstrates an urgent need to find alternative ways to improve animal health and thus decrease the application of antimicrobials. This is reflected in the main focus of current research, which has shifted from developing new therapeutic agents to working together to improve animal health, welfare, and sustainable food production. Herbs may play a crucial role in sheep nutrition, offering benefits such as enhanced feed efficiency, immune support, and improved growth. *Pimpinella anisum* has been shown to serve as an effective natural feed additive, improving feed utilization efficiency [6]. Rich in antioxidants, vitamins, and minerals, herbs strengthen immunity and enhance disease resistance. Phytobiotic additives from *Leuzea carthamoides* and *Echinacea purpurea* have been linked to increased body weight and improved livestock health [7]. Additionally, certain herbs enhance feed palatability, as seen with *Ilex paraguariensis*, which improves feed intake and wool growth [8]. Moreover, herbal preparations, plant extracts, and isolated phytoconstituents offer the possibility to modify numerous intestine functions under physiological and pathological conditions. The utility of medicinal plants to control gut functionality is also demonstrated by animal behavior. Ruminants, both wild and domestic, exhibit self-medicating behaviors, selectively foraging on plants with bioactive compounds to mitigate health challenges. Empirical evidence suggests that ruminants adaptively increase the intake of plant secondary metabolites with antiparasitic properties in response to gastrointestinal infections, thereby enhancing their overall health and fitness. Notable examples of medicinal foraging include the consumption of *Pistacia lentiscus*, *Lysiloma latisiliquum*, *Phillyrea latifolia*, and various Poaceae species, which are known for their bioactive potential in alleviating gastrointestinal distress [9,10,11]. In the case of gut dysmotilities, which often accompany infectious and invasive diseases, controlling contractility is an essential element of symptomatic therapy [12]. Therefore, it seems crucial to understand the impact of herbal products that have beneficial effects on animals towards intestine motility. Consequently, the aim of this study is to verify the impact of a standardized *Melissa officinalis* extract and some of its main constituents on the spontaneous and pharmacologically-induced contractility of sheep jejunum and colon. Furthermore, this study aims to identify specific health conditions in which the herb would be most effective as a symptomatic therapeutic agent.

## 2. Materials and Methods

### 2.1. Chemicals

A modified Krebs–Henseleit solution (MK-HS) was used for the transportation and as an incubation medium for the segments of sheep intestines. This solution was prepared by dissolving 123.76 mM NaCl, 5 mM KCl, 2.5 mM CaCl_2_, 1.156 mM MgSO_4_, 14.5 mM NaHCO_3_, 2.75 mM KH_2_PO_4_, and 12.5 mM glucose (Avantor Performance Materials, Gliwice, Poland) in distilled water. The pH of the buffer was kept at 7.35–7.45 thanks to the continuous delivery of carbogen (95% O_2_/5% CO_2_). The MK-HS temperature was maintained at 38 °C to simulate the physiological body temperature of the animal. Acetylcholine chloride (ACh) was used as a myocontractile agent, and isoproterenol hemisulfate salt (Isop) (both from Sigma-Aldrich, Darmstadt, Germany) as a relaxant control substance. The primary solution and subsequent dilutions of ACh and Isop were dissolved in the incubation buffer.

Lemon balm (*Melissa officinalis* L.) leaf extract (Nor-balm^®^) was manufactured by the company Nor-Feed^®^ (Beaucouzé, France), as described previously in [13], and standardized for the content of rosmarinic acid [14]. It was evaluated in the set of concentrations from 0.1 µg/mL to 0.1 mg/mL. The primary solutions and progressive dilutions of the extract were made using the MK-HS. Given that the main phenolic acids in lemon balm are rosmarinic acid (RA), chlorogenic acid (ChA) (Sigma-Aldrich, Darmstadt, Germany), and lithospermic acid (LA) (PhytoLab, Vestenbergsgreuth, Germany) [13], their effect was studied additionally to the trials on the extract. Each phenolic acid was tested in the range of 0.001 to 100 µM, with each acid in a separate set of experiments. The stock solutions of RA, ChA, and LA were prepared in dimethyl sulfoxide (DMSO, Sigma-Aldrich, Darmstadt, Germany) and subsequently diluted with distilled water, ensuring that the final DMSO concentration in the tissue incubation chambers did not exceed 0.5%.

### 2.2. Animals, Isolation, and Preparation of Smooth Muscle Segments

Intestinal segments were obtained from 5-month-old Uhruska sheep of both sexes (approx. 35 kg b.w.), routinely slaughtered, and subjected to ante-mortem and post-mortem examinations conducted by an official veterinarian. The experiments were performed between January and May, and the animals were slaughtered always on the day of the trial. The absence of exclusion criteria in animal selection aimed to ensure a representative population and evaluate the effects of the test substances independently of specific group selection parameters. The specimens were collected each time from at least five different sheep, both the small intestine (proximal part of the jejunum) and the colon (proximal part of the spiral colon) regions. Immediately following collection, the intestines were flushed to remove gut contents, immersed in ice-cold (0–4 °C) MK-HS, and transported to the laboratory (max. 45 min). There, the intestinal specimens were cut open longitudinally and pinned on the dish to be dissected by a scalpel blade into smaller, thin, flat, and rectangle-shaped strips (5 × 20 mm) parallel to the orientation of the longitudinal and circular smooth muscle fibers, depending on the experimental requirements.

### 2.3. The Course of Experiments

To enable the tissues’ adaptation to in vitro conditions, the samples were preincubated before every experiment for 45 min in the MK-HS at 38 °C without applying any tension. Flushing the chambers with fresh, warm MK-HS was conducted every 15 min to ensure sufficient tissue nutrients. During the preincubation period, spontaneous contractility of the smooth muscle was monitored. After 45 min of preincubation, tension (0.01 N) was carefully applied to all tested muscle strips for 15 min, after which the tension (0.01 N) was increased another time. Strips reacting properly to ACh (1 µM) twice and exhibiting distinct and rhythmic spontaneous contractility were employed for the trial. The solvent of the test substance was administered before the actual experiment, i.e., MK-HS for the lemon balm extract and DMSO (0.5%) for all three acids (negative control). Following buffer exchange, the tissue strips were exposed to ACh (10 µM), and motor activity was recorded for 5 min. The medium was then flushed and, after stabilization of spontaneous contractility, either the extract or one of the active compounds was introduced. The response was monitored for 5 min, after which another dose of ACh (10 µM) was applied. Five minutes later, the buffer was replaced, and treatment with a higher concentration of the test substance was initiated. This procedure allowed for the non-cumulative assessment of the extract and three acids on both spontaneous and ACh-induced contractility. The experiment concluded with the administration of ACh and isoproterenol to confirm intestinal reactivity and vitality.

### 2.4. Data Registration and Expression

Specimens were placed in the incubation chambers of 5 mL each, filled with the MK-HS, and saturated with carbogen (95% O_2_ and 5% CO_2_). Five to six strips from five to six different animals were suspended separately in individual chambers. The preparations were placed parallel to the orientation of the longitudinal or circular smooth muscle fibers, depending on the experimental requirements, and fixed with one end (distal) attached to a steel hook on a tissue holder and the other (proximal) to a silk thread connected to an isotonic force transducer (F30, type 372, Hugo Sachs Elektronik, March-Hugstetten, Germany). During the experiment, the specimens’ reactivity was measured for tension changes by using a bridge amplifier (DBA, type 660, Hugh Sachs Elektronik, March-Hugstetten, Germany). The transducer apparatus was connected to an analog–digital registration set (PowerLab, ADInstruments, Sydney, Australia). The motor activity of the samples was recorded by the Chart v7.0 program (ADInstruments, Sydney, Australia). Data analysis was performed using Chart v8.1 programs (ADInstruments, Sydney, Australia) and Microsoft Office Excel. All generated data went through critical statistical analysis.

### 2.5. Statistical Analysis

The impact of all tested substances was determined by measuring alterations in the tension of the smooth muscle preparations 5 min before and after applying any substance, quantified as the area under the curve (AUC). All results are presented as a percentage of the control reaction, defined by the intestine’s response to MK-HS or DMSO (0.5%) in the case of spontaneous activity analysis, and smooth muscle contraction induced by ACh (10 µM) at the start of the reactivity analysis, respectively. Reactions to MK-HS, DMSO (0.5%), and acetylcholine at control doses were stated at 100% (control). Results are expressed as mean values from a minimum of five replicates of experiments. Statistical analysis was performed using one-way ANOVA through TIBCO StatSoft, Inc. (Tulsa, OK, USA, 2019), STATISTICA (data analysis software system), version 13.3 (https://www.statsoft.pl/statistica-i-tibco-software/, accessed on 10 January 2025). The Dunnett test was used for pairwise comparisons, with the effects of MK-HS, DMSO (0.5%), and ACh (10 µM) in DMSO (0.5%) serving as references. A *p*-value of <0.05 was considered statistically significant.

## 3. Results

### 3.1. The Effect of the Standardized Melissa officinalis Extract on the Spontaneous Motoric Activity of Ovine Jejunum and Colon Specimens

**Figure 1:**
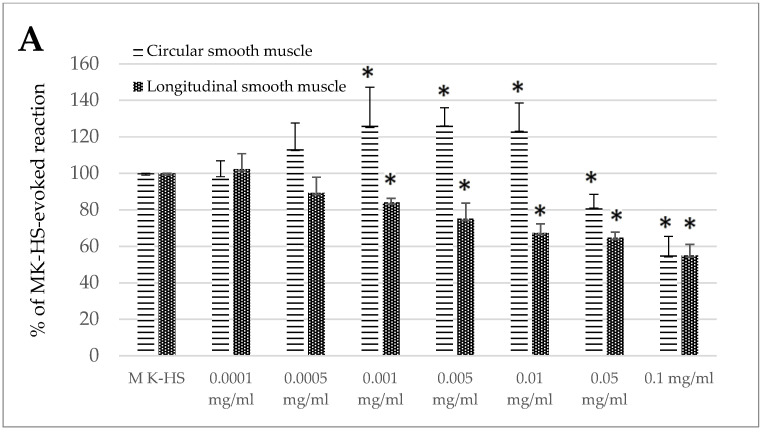

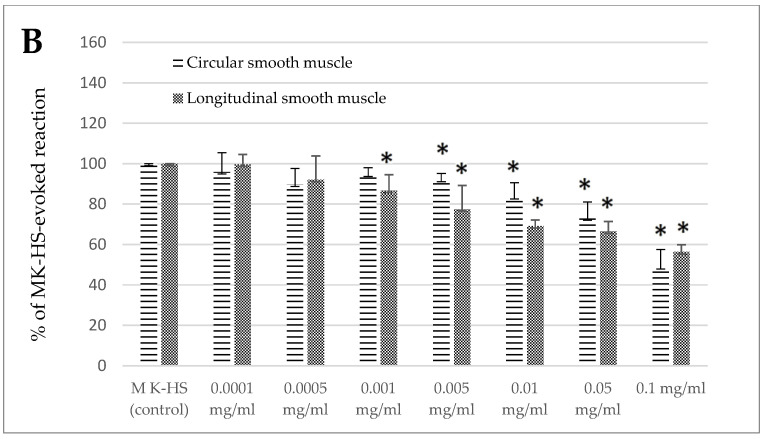
The effect of the *Melissa officinalis* extract on the spontaneous contractility of ovine jejunum (**A**) and colon (**B**)—circular and longitudinal smooth muscle. The results are presented as a percentage of spontaneous activity relative to the control (solvent). Data represent the mean ± standard deviation from six independent experiments. * *p* ≤ 0.05 vs. MK-HS.

The effects of the standardized extract of *Melissa officinalis* were dose-dependent (Figure 1A,B). A fascinating biphasic response was observed for the jejunum circular smooth muscle, where the reaction was myocontractile in the concentration from 0.001 to 0.01 mg/mL, ranging from 126.11 ± 21.10 to 124.11 ± 14.40% of the control reaction. However, when administered in higher concentrations (0.05 and 0.1 mg/mL), the effect was myorelaxant, ranging from 81.97 ± 6.48 to 55.16 ± 10.31% of the control response (Figure 1A). In the case of the jejunum longitudinal smooth muscle and both types of colon segments, the reaction provoked by lemon balm was myorelaxant. The size of the response observed in the longitudinal smooth muscle of the jejunum came to 84.17 ± 2.16 and 55.02 ± 6.03% of the control reaction for the application of the extract in the concentration of 0.001 and 0.1 mg/mL, respectively (Figure 1A). In the case of the colon, the effect was similar in circular and longitudinal smooth muscle (Figure 1B). The use of the extract resulted in the reduction of spontaneous contractility. The myorelaxant effect was significant when lemon balm was applied in the concentration of 0.005 mg/mL or higher. and reached from 92.10 ± 3.05% (0.005 mg/mL) up to 48.88 ± 8.60% (0.1 mg/mL) of the control reaction for circular muscle. A notable decrease in the intensity of the spontaneous activity in colon longitudinal smooth muscle preparations was measured if lemon balm was used at the concentration of 0.001 mg/mL or higher, and then reached from 86.72 ± 7.76% (0.001 mg/mL) up to 56.34 ± 3.53% (0.1 mg/mL) of the control reaction (Figure 1B).

### 3.2. The Effect of RA, ChA, and LA on the Spontaneous Motoric Activity of Ovine Jejunum and Colon Specimens

**Figure 2:**
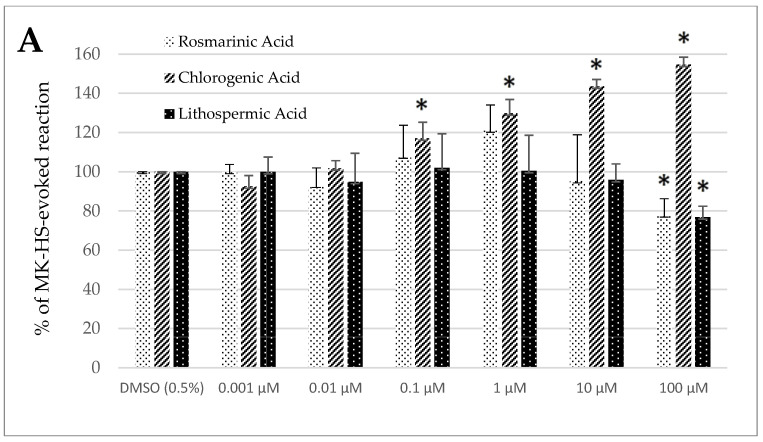

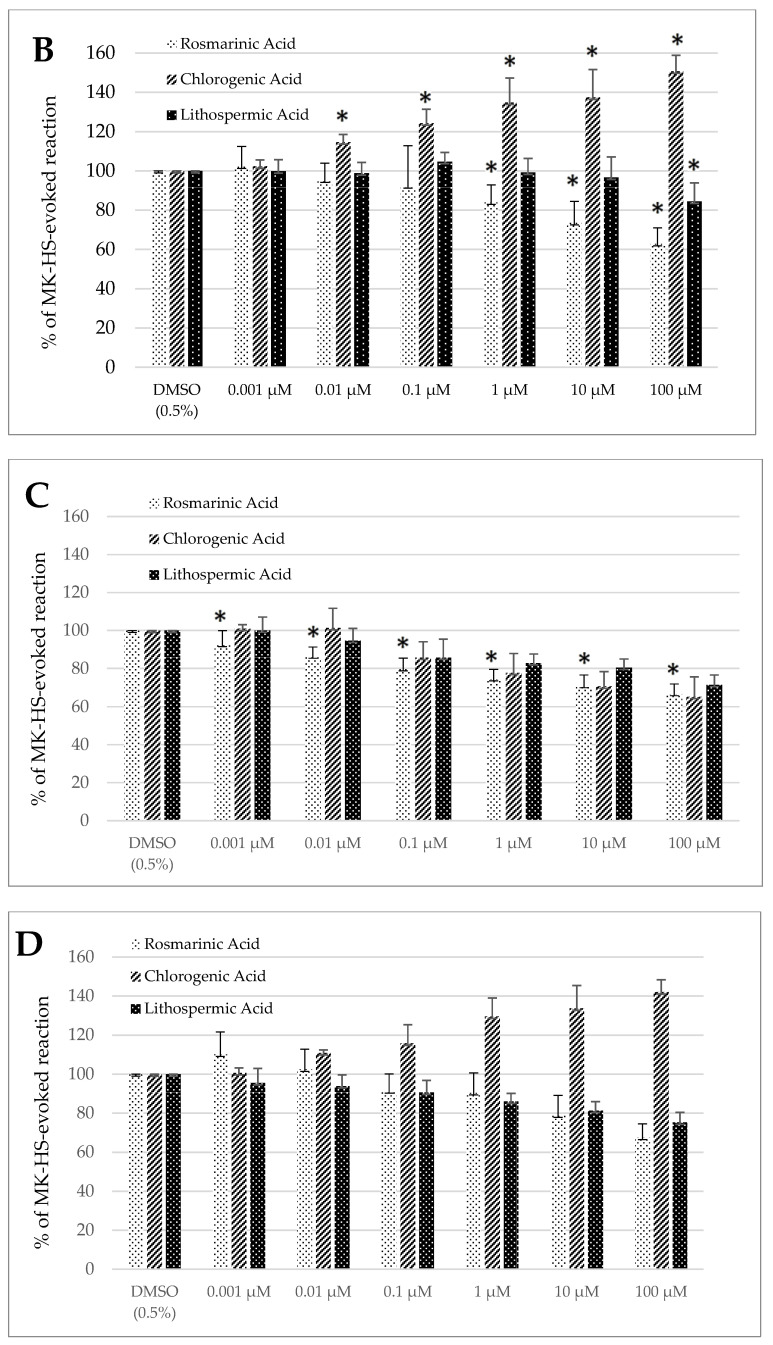
The effect of rosmarinic, chlorogenic, and lithospermic acids, on the spontaneous contractility of ovine jejunum (**A**,**B**) and colon (**C**,**D**)—circular (**A**,**C**) and longitudinal (**B**,**D**) smooth muscle. The results are presented as a percentage of spontaneous activity relative to the control (solvent). Data represent the mean ± standard deviation from five independent experiments. * *p* ≤ 0.05 vs. DMSO (0.5%).

Control treatment with the solvent, DMSO (0.5%), generated no significant change in the spontaneous contractility of the sheep’s longitudinal and circular jejunum and colon segments (Figure 2A–D).

In the case of rosmarinic acid, a remarkable modification of the spontaneous contractility was noticed in the jejunum circular smooth muscle and the colon segments of both origins; the reaction was always myorelaxant. The decrease of spontaneous contractility in the jejunum circular specimens was significant only when RA was used in the highest tested concentration (100 µM) and came to 77.86 ± 8.38% of the control reaction. By contrast, in the case of longitudinal smooth muscle preparations, the change ranged from 84.00 ± 8.80% (1 µM) to 62.85 ± 8.11% (100 µM) of the control reaction (Figure 2A,B). The analysis of the motoric patterns of the colon smooth muscle showed a decrease in spontaneous contractility, which ranged from 92.50 ± 7.44% (0.001 µM) to 66.74 ± 5.23% (100 µM) and from 78.87 ± 10.28% (10 µM) to 67.44 ± 7.06% (100 µM) of the control reaction for the circular and longitudinal smooth muscle preparations, respectively (Figure 2C,D).

In the case of chlorogenic acid, the effect was myocontractile, except for the colon’s circular smooth muscle. Remarkable changes were observed when the ChA was administered at 0.1 µM or higher concentration in the jejunum circular smooth muscle. The size of the response induced by ChA ranged from 117.07 ± 8.13% (0.1 µM) to 154.76 ± 3.75% (100 µM) of the control reaction in the jejunum longitudinal smooth muscle and from 114.55 ± 3.96% (0.01 µM) to 150.73 ± 8.10% (100 µM) of the control treatment in the jejunum circular smooth muscle (Figure 2A,B). A similar effect was observed in colon preparations consisting of longitudinally oriented fibers. ChA induced myocontractile reaction, which force ranged from 110.87 ± 1.52% (0.01 µM) to 142.06 ± 6.26% (100 µM) of the control treatment with DMSO (0.5%) (Figure 2D). In the case of the colon circular smooth muscle specimens, the effect was opposite, i.e., myorelaxant. The magnitude of changes in spontaneous contractility varied from 85.74 ± 8.32% (0.1 µM) to 65.17 ± 10.46% (100 µM) of the control reaction (Figure 2C).

Lithospermic acid provoked a significant decrease in the spontaneous motor activity of both the jejunum and the colon smooth muscle. In the case of the jejunum smooth muscle, the effect was observed only when LA was used in the highest concentration i.e., 100 µM. It amounted then to 76.89 ± 5.51% and 84.49 ± 9.41% of the response to DMSO (0.5%) for circular longitudinal smooth muscle strips, respectively (Figure 2A,B). In the case of the colon segments consisting of circularly oriented fibers, the size of inhibited contractility ranged from 85.81 ± 9.65% to 71.52 ± 5.02% of the control treatment for LA applied in a dose of 0.1 and 100 µM, respectively (Figure 2C). The reduction of motility patterns in the colon longitudinal strips was significant if LA was used at the concentration of 0.1 µM and greater, and dropped at the maximum dose (100 µM) to 75.29 ± 5.13% of the control reaction (Figure 2D).

### 3.3. The Effect of the Standardized Melissa officinalis Extract on Acetylcholine-Induced Contractility of Ovine Jejunum and Colon Specimens

**Figure 3:**
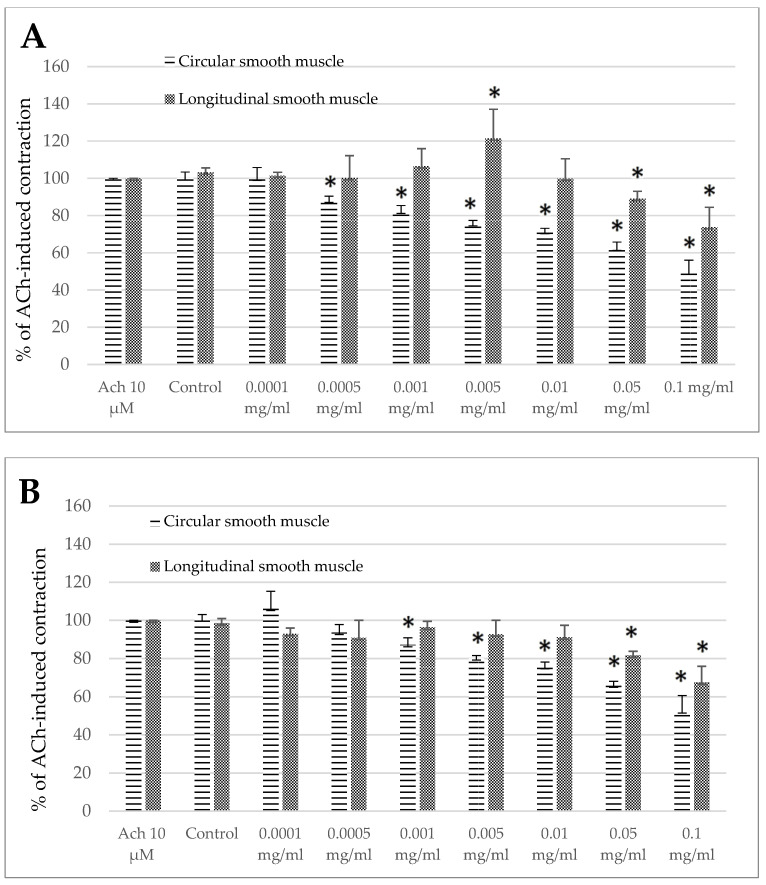
The effect of the *Melissa officinalis* extract on the ACh-evoked reaction of ovine jejunum (**A**) and colon (**B**)—circular and longitudinal smooth muscle. The data are expressed as a percentage of ACh-induced contraction, which is defined as 100%. The control reaction (Control) represents the response of the strips to ACh dissolved in MK-HS. Data represent the mean ± standard deviation from 6 independent experiments. * *p* ≤ 0.05 vs. ACh.

The standardized *Melissa officinalis* extract had clear antispasmodic effects towards the ovine jejunum and colon segments (Figure 3A,B). The only exception was when it was applied in a specific concentration of 0.005mg/mL in the jejunum longitudinal smooth muscle. In this situation, using lemon balm significantly increased ACh-induced contractions. The measured reaction came to 121.39 ± 15.69% of the control response to Ach (Figure 3A). However, even in the case of the jejunum longitudinal segments, the application of the *Melissa officinalis* extract at higher concentrations resulted in a clear myorelaxant effect. The extract reduced the response to ACh to 89.14 ± 3.85% and 73.84 ± 10.56% of the control treatment when used in a dose of 0.05 and 0.1mg/mL, respectively (Figure 3A).

In the jejunum preparations consisting of circular smooth muscle fibers, a notable reduction of ACh-induced contraction was provoked by the *Melissa* extract used in the concentration range of 0.0005–0.1 mg/mL, reaching the maximum decrease to the level of 49.35 ± 6.69% of the control reaction to ACh (Figure 3A). In the case of the colon circular smooth muscle, remarkable alteration of ACh-induced contraction was registered when the extract was used in a concentration of 0.001 mg/mL or higher, amounting to 87.20 ± 3.70% and a maximum of 52.53 ± 8.18% of the control reaction, for the extract used in the concentration of 0.001mg/mL and 0.1mg/mL, respectively (Figure 3B). Noteworthy, the colon specimens containing longitudinal smooth muscle turned out to be less susceptible to the *Melissa* extract than the circular oriented fibers. The range of effective concentrations of the lemon balm extract for the colon longitudinal smooth muscle was between 0.05 and 0.1 mg/mL, and the size of the myorelaxant amounted then to 81.66 ± 2.11% and 67.56 ± 8.34% of the control reaction, respectively (Figure 3B).

### 3.4. The Effect of Phenolic Acids on Acetylcholine-Induced Contractility of Ovine Jejunum and Colon Specimens

**Figure 4:**
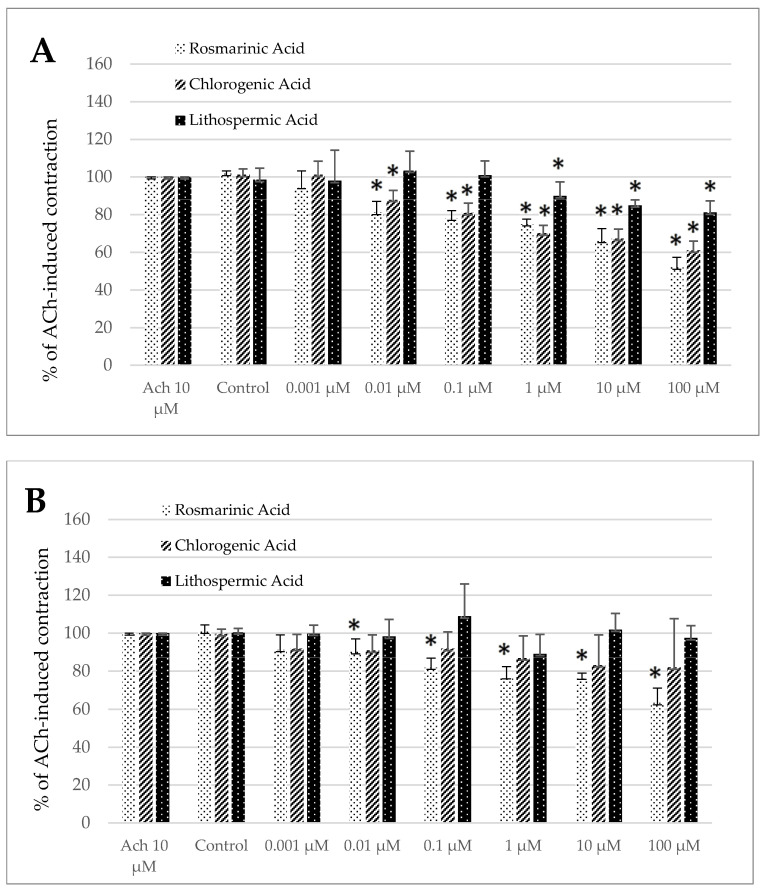

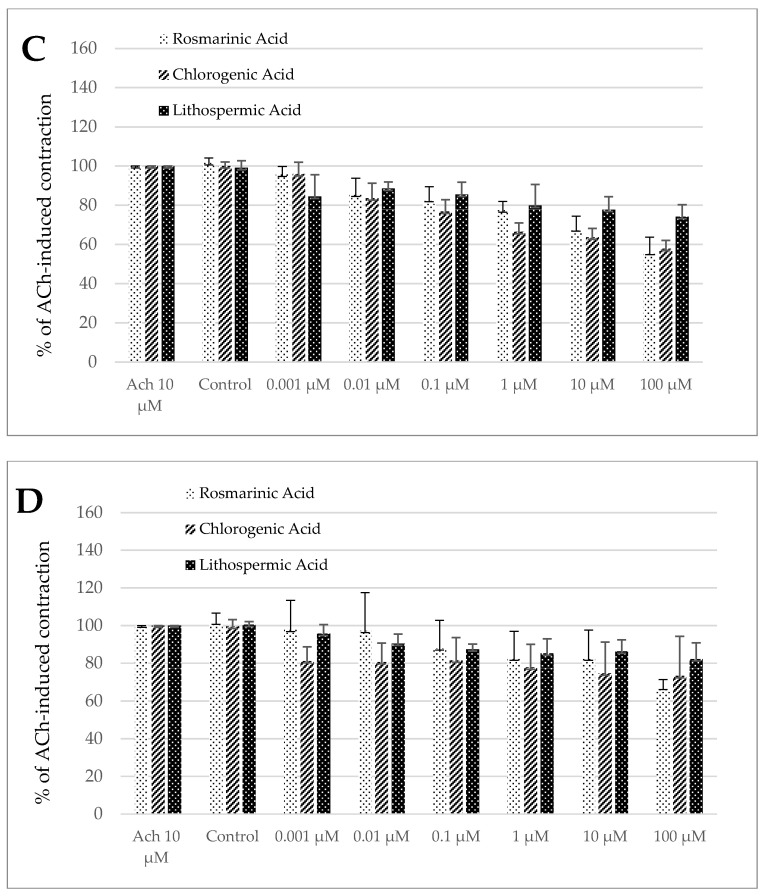
The effect of rosmarinic, chlorogenic, and lithospermic acids on the spontaneous contractility of ovine jejunum (**A**,**B**) and colon (**C**,**D**)—circular (**A**,**C**) and longitudinal (**B**,**D**) smooth muscle. The data are expressed as a percentage of ACh-induced contraction, which is defined as 100%. The control reaction (Control) represents the response of the strips to ACh dissolved in DMSO. Data represent the mean ± standard deviation from five independent experiments. * *p* ≤ 0.05 vs. ACh.

Noteworthy, the control treatment with the solvent (DMSO 0.5%) produced a significant change in the size of ACh-induced contractility neither in the jejunum nor in the colon preparation (Figure 4A–D) of both longitudinal and circular segments.

Rosmarinic acid notably influenced the ACh-induced contractility in both segment types and muscle layers (Figure 4A–D). Regardless of the strip origin, the observed effect was antispasmodic and statistically significant across a broad concentration range. For the jejunum specimens, the reduction in contractile activity reached a magnitude of 81.06 ± 5.91% and 51.97 ± 5.33% for the circular smooth muscle and 90.12 ± 6.85% and 63.41 ± 7.74% for the longitudinal smooth muscle of the control reaction for RA used in the concentration of 0.01 and 100 µM, respectively (Figure 4A,B). In the case of the colon specimens, the antispasmodic reaction was noteworthy in the range of concentration from 0.01 to 100 µM in the circular smooth muscle and from 1 to 100 µM in the longitudinal smooth muscle. The maximum inhibition of ACh-induced concentration was recorded when RA was applied in the highest dose and amounted then to 55.79 ± 7.95% and 67.12 ± 4.20% of the control ACh treatment in the colon circular and longitudinal smooth muscle strips, respectively (Figure 4C,D).

For chlorogenic acid only in the jejunum longitudinal strips, no significant changes in ACh-induced contractions were observed (Figure 4B). However, in all other cases (the jejunum circular smooth muscle and both types of the colon specimens), ChA provoked a notable decrease in ACh-induced contractions. In the circular smooth muscle of the jejunum, a pronounced reduction in the response was observed when ChA was applied at concentrations of 0.01 µM or greater. The magnitude of the resulting reaction varied from 88.00 ± 4.90% (0.01 µM) to 61.01 ± 4.99% (100 µM) the control treatment (Figure 4A). While in the colon circular strips, the effective doses started with 0.01 µM. The magnitude of ACh-induced contraction was decreased up to 83.62 ± 7.63% and even 57.82 ± 4.20% of the control reaction for ChA applied in the concentration of 0.01 and 100 µM, respectively (Figure 4C). Finally, in the case of the colon longitudinal smooth muscle, the reaction induced by ChA was significant in the concentration range of 0.001 to 100 µM and amounted to 81.17 ± 7.54% and even 73.26 ±21.03% of the control reaction, respectively (Figure 4D).

Eventually, lithospermic acid provoked a similar change in contractility to chlorogenic acid, i.e., only the jejunum longitudinal strips did not respond to LA. By contrast, in all other types of muscle strips, LA provoked a remarkable decrease in ACh-induced contractions. For the jejunum circular smooth muscle preparations, a significant decrease of ACh-evoked contraction was measured in the presence of LA in the concentration range of 1–100 µM and ranged respectively from 89.89 ± 7.55% to 81.21 ± 6.04% of the control reaction (Figure 4A). In the case of the colon preparation, in circular smooth muscle LA provoked a decreased reaction to ACh, which came to 84.41 ± 11.15% and 74.20 ± 6.03% of the control contraction for the acid utilized in the concentration of 0.001 and 100 µM, respectively (Figure 4C). In the case of the colon longitudinal smooth muscle, a significant decrease in ACh-induced contraction was observed in the presence of LA in the concentration of 0.01 µM and higher. The maximum inhibition of ACh-provoked reaction was detected for LA used in the highest dose (100 µM) and amounted to 82.21 ± 8.63% of the control ACh treatment (Figure 4D).

## 4. Discussion

As a feed additive, *Melissa officinalis* offers various benefits for animal welfare. The experiment on Arabi sheep indicated that the high levels of added *melissa* leaves to feed significantly benefited productive traits (body weight, weight gain, feed consumption, and conversion factor) [15]. When selecting herbs to add to the feed, not only the plants’ properties but the animals’ preferences should be taken into account. It has been noticed that lemon balm is tasty for pigs, cows, and chickens [16]; it suggests that *Melissa officinalis* might also be palatable to sheep and, as a highly palatable herb, may increase feed and nutrient intake.

The safety of a commercially available dried aqueous ethanol extract of *Melissa officinalis* L. leaves (Nor-Balm^®^, Beaucouzé, France) was validated as a feed additive for all animal species. The maximum proposed use level is 100 mg/kg of complete feed. No safety concern arises for the consumer from animal feed [13]. Polish research has confirmed the positive effect of the moderate administration of mixtures of herbs, including lemon balm, on the flavor of meat lamb [17]. Since the extract is always applied orally, it has the chance to modulate the functioning of the digestive tract. Therefore, it is important to investigate and understand its potential to affect gut motility. Gastrointestinal disorders might seriously threaten the animal’s welfare and cause financial losses for the farmer. Moreover, gastrointestinal disorders impacting motility play a critical role in digestion, nutrient absorption, and the maintenance of gut microbiome balance [18,19]. One of the most common diseases of the digestive tract in sheep is infection caused by *Clostridium perfringens*, which can occur mainly in the case of a weakened intestinal peristalsis. *Clostridium perfringens* is a regular component of the intestinal microbiota of sheep, which occurs in more significant quantities in the ileum [20,21]. In the case of a weakened peristalsis and an inappropriate diet, a pathological, excessive multiplication of this bacterium is likely to happen. Considering the results of our experiments (and assuming positive verification in in vivo trials), administering a feed additive with lemon balm to a healthy animal may reduce intestinal peristalsis, which increases the risk of infection with *Clostridium perfringens*. Therefore, lemon balm should not be a permanent feed additive, especially if added in large quantities. Still, in the case of illness, if one of the main symptoms is diarrhea, lemon balm should have a therapeutic effect, limiting intestinal hypercontractility. It can also improve the palatability of feed when appetite is reduced during illness.

The comprehensive understanding of the effects of lemon balm on critical tissues and their functionality in various animal species remains insufficiently explored. The trials’ outcomes prove to undouble the significant and potent effect of lemon balm and its phenolic acids on modifying sheep GIT motility (Table 1). Regarding spontaneous contractions, the *Melissa officinalis* extract, rosmarinic acid, and lithospermic acid induced a significant decrease in spontaneous contractility in the sheep jejunum and colon. By contrast, chlorogenic acid exhibited myocontractile effects except for the colon circular smooth muscle preparations, where the effect was myorelaxant, as with the other acids. The results show a decrease in spontaneous activity after administration of the tested substances, so the *Melissa officinalis* extract should not be used for hypomotility or even atony of GIT in sheep. In the case of ACh-induced contractions, the *Melissa officinalis* extract and all tested phenolic acids decreased significantly acetylcholine-induced contractions in the sheep longitudinal and circular smooth muscle strips of the colon and jejunum (except for chlorogenic and lithospermic acid in the longitudinal strips of the jejunum, which did not cause significant impact). The observation of the antispasmodic effect could potentially benefit symptomatic therapy of sheep experiencing hypermotility disorders, demonstrated mainly as diarrhea, and it might help eliminate painful intestinal cramps and slow down peristalsis. Given the convergent effects of the whole lemon balm extract and its phenolic acids, investigating their concurrent administration could provide insight into potential synergistic or hyperadditive interactions, enhancing their applicability in clinical settings. However, it is crucial to acknowledge that the findings presented were derived from ex vivo conditions. Alternative research methods help minimize the use of animals in in vivo studies, reducing research costs, duration, and supporting animal welfare. Due to the complexity and variability of rumen fermentation in vivo, these results should be interpreted cautiously. Furthermore, there is currently no experimental evidence on the metabolic fate of the lemon balm extract in the ruminant gastrointestinal tract, nor assurance that phenolic acids reach sufficient intestinal concentrations to elicit the observed effects. An additional factor influencing the bioavailability and activity of the phenolic acids is their potential interaction with other feed components, particularly proteins [22], and their impact on methanogenesis [23].Given the interspecies variations in the anatomical structure of the digestive tract, dietary composition, housing conditions, and the intended use of animals, research should be very carefully extrapolated to other species. Nevertheless, there are examples of using sheep as a large animal model for investigating and treating human disorders [24], or as a representative for other ruminants [25,26]. Nevertheless, it is beneficial to conduct specific research across different animal species whenever possible to avoid misinterpretation [27,28]. The records of trials with *Melissa officinalis* and its ingredients demonstrate that the effects may indeed vary qualitatively and quantitatively between animal species. When comparing the results presented herein, it was found that *Melissa officinalis* also decreased the frequency of spontaneous contractions on the jejunum of the mice [29], the ileum of rats [30], and the jejunum of chicken [14]. However, in the case of swine, the effect is the opposite. Lemon balm extract caused a progressive increase in the spontaneous contractility of the jejunum and colon longitudinal smooth muscle, and rosmarinic and lithospermic acids provoked dose-dependently increased colon motility [31]. The contradictory results obtained in swine tissue might result from a more dominant role of phenolic acids other than active constituents, e.g., essential oil ingredients. On the other hand, the third of the phenolic acid tests, i.e., chlorogenic acid, was found to cause myorelaxation in the thoracic aorta smooth muscle of rats [32].

Interestingly, the effects are similar when comparing the research on the effects of *Melissa officinalis* to other plants of similar phytochemical profiles. For example, in an experiment involving a volatile oil of *Rosmarinus officinalis* leaves (rich in rosmarinic acid) on the vascular smooth muscle of a rabbit, the effect was also myorelaxant [33]. In an experiment involving sheep abomasal preparations [34], essential oil from *Artemisia dracunculus* (rich in chlorogenic acid) provoked complete relaxation of the spontaneous activity and reduction of the force of ACh-induced contractions [35]. Moreover, *Crataegus gracilior,* a plant rich in chlorogenic acid, was also observed to relax the smooth muscle in rat-isolated aorta rings [36].

The influence of lemon balm and phenolic acids on the motor activity of the jejunum and colon may significantly impact the functioning of the sheep gut. A strong influence on intestinal motility could lead to alterations in digestion time, peristalsis, nutrients and antigens absorption, and microbiota homeostasis through the gastrointestinal tract. This study indicates that the effect of *Melissa officinalis* and some of its significant constituents may play an essential role in controlling the contractility of the sheep jejunum and colon. By offering an herbal-based approach to gut health and motility regulation, this research contributes to reducing antibiotic and synthetic drug use in sheep farming, aligning with global efforts to combat antimicrobial resistance. These findings provide a foundation for further research into the development of herbal supplements or medications targeting gut motility disorders in sheep.

## 5. Conclusions

The results unequivocally demonstrate that both the *Melissa officinalis* extract and its primary phenolic constituents exert a myorelaxant effect on the smooth muscle of the sheep jejunum and colon under ex vivo conditions. This study underscores the significant potential of the *Melissa officinalis* extract as a feed additive to modulate intestinal motility in sheep, addressing various challenges in livestock production. Further in vivo investigations are necessary to validate these preliminary findings and elucidate the specific mechanisms through which *Melissa officinalis* influences these critical gastrointestinal functions.

## Figures and Tables

**Table 1:** A summary of the effects of the *Melissa officinalis* extract and three phenolic acids on ovine intestine motoric activity: (**A**)—spontaneous contractility; (**B**)—ACh-induced contractility.

(**A**)				
	**GIT Section**			
**Muscle Fiber**	**Jejunum**		**Colon**	
	**Circular**	**Longitudinal**	**Circular**	**Longitudinal**
**Lemon Balm extract**	R	R	R	R
**Rosmarinic Acid**	R	R	R	R
**Chlorogenic Acid**	C	C	R	C
**Lithospermic Acid**	R	R	R	R
(**B**)				
	**GIT Section**			
**Muscle Fiber**	**Jejunum**		**Colon**	
	**Circular**	**Longitudinal**	**Circular**	**Longitudinal**
**Lemon Balm extract**	R	R	R	R
**Rosmarinic Acid**	R	R	R	R
**Chlorogenic Acid**	R	X	R	R
**Lithospermic Acid**	R	X	R	R

C—contraction of the smooth muscle, R—relaxation of the smooth muscle, X—no significant effect.

## Data Availability

The original contributions presented in this study are included in the article. Further inquiries can be directed to the corresponding author.

## Abbreviations

ACh	Acetylcholine chloride
ChA	Chlorogenic acid
DMSO	Dimethyl sulfoxide
GI	Gastrointestinal
GIT	Gastrointestinal tract
Isop	Isoproterenol
LA	Lithospermic acid
MK-HS	Modified Krebs–Henseleit solution
RA	Rosmarinic acid

## References

[1] Pulina G., Milán M.J., Lavín M.P., Theodoridis A., Morin E., Capote J., Thomas D.L., Francesconi A.H.D., Caja G. (2018). Invited Review: Current Production Trends, Farm Structures, and Economics of the Dairy Sheep and Goat Sectors. J. Dairy Sci..

[2] Tirado R. (2009). Defining Ecological Farming.

[3] (2021). EFSA The European Union Summary Report on Antimicrobial Resistance in Zoonotic and Indicator Bacteria from Humans, Animals and Food in 2018/2019. EFSA J..

[4] Singer R.S., Porter L.J., Thomson D.U., Gage M., Beaudoin A., Wishnie J.K. (2019). Raising Animals Without Antibiotics: U.S. Producer and Veterinarian Experiences and Opinions. Front. Vet. Sci..

[5] Gernat A.A., Santos F.B.O., Grimes J.L. (2021). Alternative Approaches to Antimicrobial Use in the Turkey Industry: Challenges and Perspectives. Ger. J. Vet. Res..

[6] Kour D., Sharma V.K., Sharma R.K., Pathak A.K., Rastogi A. (2023). Evaluation of Native Medicinal Plants as Feed Additives in the Sheep Ration. Indian J. Anim. Sci..

[7] Saparova E., Zubova T. (2019). The Effectiveness of Phytobiotic Additives in the Diet of Sheep. IOP Conf. Ser. Earth Environ. Sci..

[8] Celi P., Raadsma H.W. (2009). The effects of Yerba Mate (*Ilex paraguarensis*) supplementation on the productive performance of lambs. Ruminant Physiology.

[9] Amit M., Cohen I., Marcovics A., Muklada H., Glasser T.A., Ungar E.D., Landau S.Y. (2013). Self-Medication with Tannin-Rich Browse in Goats Infected with Gastro-Intestinal Nematodes. Vet. Parasitol..

[10] Gradé J.T., Tabuti J.R.S., Van Damme P. (2008). Four Footed Pharmacists: Indications of Self-Medicating Livestock in Karamoja, Uganda. Econ. Bot..

[11] Villalba J.J., Miller J., Ungar E.D., Landau S.Y., Glendinning J. (2014). Ruminant Self-Medication against Gastrointestinal Nematodes: Evidence, Mechanism, and Origins. Parasite.

[12] Singh R., Zogg H., Wei L., Bartlett A., Ghoshal U.C., Rajender S., Ro S. (2021). Gut Microbial Dysbiosis in the Pathogenesis of Gastrointestinal Dysmotility and Metabolic Disorders. J. Neurogastroenterol. Motil..

[13] Posłuszny M.A., Chłopecka M., Suor-Cherer S., Cisse S., Benarbia M.e.A., Mendel M. (2023). Modulation of Chicken Gut Contractility by Melissa Officinalis—Ex Vivo Study. Poult. Sci..

[14] Bampidis V., Azimonti G., Bastos M.d.L., Christensen H., Kouba M., Kos Durjava M., López-Alonso M., López Puente S., Marcon F., Mayo B. (2020). Safety and Efficacy of a Dried Aqueous Ethanol Extract of *Melissa officinalis* L. Leaves When Used as a Sensory Additive for All Animal Species. EFSA J..

[15] Wedah H.A., Mohamed A.S., Al-Yassiri A.J. (2020). Effect of Added Different Levels of Melissa Leaves Powder to Diet on Some Productive Traits of Arabi Sheep. Plant Arch..

[16] Grela E., Klebaniuk R., Kwiecien M., Pietrzak K. (2013). Fitobiotyki w produkcji zwierz˛ecej [Phytobiotics in animal production]. Prz. Hod..

[17] Bodkowski R., Patkowska-Sokoła B., Szmatko T. (1992). Wpływ dodatku naturalnych biostymulatorów na użytkowość mięsna jagniąt oraz opłacalność tuczu. Biul. Inf. Przem. Pasz..

[18] Lee B., Bello-Pérez L.A., Lin A.H., Kim C.Y., Hamaker B.R. (2013). Importance of Location of Digestion and Colonic Fermentation of Starch Related to Its Quality. Cereal Chem..

[19] Sensoy I. (2021). A Review on the Food Digestion in the Digestive Tract and the Used In Vitro Models. Curr. Res. Food Sci..

[20] Uzal F.A., Songer J.G. (2008). Diagnosis of Clostridium Perfringens Intestinal Infections in Sheep and Goats. J. Vet. Diagn. Investig..

[21] Mohammadabadi M.R. (2017). Role of Clostridium Perfringens in Pathogenicity of Some Domestic Animals. J. Adv. Agric..

[22] Schefer S., Oest M., Rohn S. (2021). Interactions between Phenolic Acids, Proteins, and Carbohydrates—Influence on Dough and Bread Properties. Foods.

[23] Liu Y., Li X., Diao Q., Ma T., Tu Y. (2024). In Silico and in Vitro Studies Revealed That Rosmarinic Acid Inhibited Methanogenesis via Regulating Composition and Function of Rumen Microbiota. J. Dairy Sci..

[24] Banstola A., Reynolds J.N.J. (2022). The Sheep as a Large Animal Model for the Investigation and Treatment of Human Disorders. Biology.

[25] Delano M.L., Mischler S.A., Underwood W.J. (2002). Biology and Diseases of Ruminants: Sheep, Goats, and Cattle. Laboratory Animal Medicine.

[26] Hackmann T.J., Spain J.N. (2010). Invited Review: Ruminant Ecology and Evolution: Perspectives Useful to Ruminant Livestock Research and Production. J. Dairy Sci..

[27] Karasov W.H., Douglas A.E. (2013). Comparative Digestive Physiology. Compr. Physiol..

[28] Mahayri T.M., Fliegerová K.O., Mattiello S., Celozzi S., Mrázek J., Mekadim C., Sechovcová H., Kvasnová S., Atallah E., Moniello G. (2022). Host Species Affects Bacterial Evenness, but Not Diversity: Comparison of Fecal Bacteria of Cows and Goats Offered the Same Diet. Animals.

[29] Aubert P., Guinobert I., Guilbot A., Dubourdeaux M., Neunlist M. (2016). Antispasmodic and Spasmolytic Activity of Melissa Officinalis EPS upon Mice Gastrointestinal Tract: An Ex Vivo Pilot Study. Planta Med..

[30] Sadraei H., Ghannadi A., Malekshahi K. (2003). Relaxant Effect of Essential Oil of Melissa Officinalis and Citral on Rat Ileum Contractions. Fitoterapia.

[31] Posłuszny M., Szadkowska D., Chłopecka M., Suor-Cherer S., Benarbia M.e.A., Mendel M. (2021). Melissa Officinalis as Gut Contractility Modifier in Swine—Ex Vivo Study. Planta Med..

[32] Tom E.N.L., Girard-Thernier C., Demougeot C. (2016). The Janus Face of Chlorogenic Acid on Vascular Reactivity: A Study on Rat Isolated Vessels. Phytomedicine.

[33] Aqel M.B. (1992). A Vascular Smooth Muscle Relaxant Effect of Rosmarinus Officinalis. Int. J. Pharmacogn..

[34] Jalilzadeh-Amin G., Maham M., Dalir-Naghadeh B., Kheiri F. (2011). In Vitro Effects of Artemisia Dracunculus Essential Oil on Ruminal and Abomasal Smooth Muscle in Sheep. Comp. Clin. Pathol..

[35] Ekiert H., Świątkowska J., Knut E., Klin P., Rzepiela A., Tomczyk M., Szopa A. (2021). Artemisia Dracunculus (Tarragon): A Review of Its Traditional Uses, Phytochemistry and Pharmacology. Front. Pharmacol..

[36] Hernández-Pérez A., Bah M., Ibarra-Alvarado C., Rivero-Cruz J., Rojas-Molina A., Rojas-Molina J., Cabrera-Luna J. (2014). Aortic Relaxant Activity of Crataegus Gracilior Phipps and Identification of Some of Its Chemical Constituents. Molecules.

